# Analysis of clinical factors associated with Kampo formula-induced pseudoaldosteronism based on self-reported information from the Japanese Adverse Drug Event Report database

**DOI:** 10.1371/journal.pone.0296450

**Published:** 2024-01-02

**Authors:** Kazushi Uneda, Yuki Kawai, Akira Kaneko, Takumi Kayo, Shuichiro Akiba, Tomoaki Ishigami, Hiromi Yoshida-Komiya, Masao Suzuki, Tadamichi Mitsuma

**Affiliations:** 1 Department of Kampo Medicine, Aizu Medical Center, Fukushima Medical University, Fukushima, Aizuwakamatsu, Japan; 2 Department of Medical Science and Cardiorenal Medicine, Yokohama City University Graduate School of Medicine, Yokohama, Japan; 3 Center for Gender-Specific Medicine, Fukushima Medical University, Fukushima, Japan; Teikyo University, School of Medicine, JAPAN

## Abstract

Drug-induced pseudoaldosteronism is a typical adverse effect of Kampo formulas. Previous research described the potential risks of Kampo formula-linked pseudoaldosteronism. However, few studies assessed the risk factors using a real-world database and a data-mining approach. Using the Japanese Adverse Drug Event Report database, we extracted pseudoaldosteronism reports for 148 Kampo formulas covered by Japanese national health insurance. Adverse events were decided according to the preferred terminology of the Medical Dictionary for Regulatory Activities/Japanese version 25.1. We calculated reporting odds ratio (RORs) and identified Kampo formulas as suspected causes of pseudoaldosteronism. Moreover, we evaluated clinical factors associated with Kampo formula-induced pseudoaldosteronism via logistic regression. From April 2004 to November 2022, 6334 adverse events related to the Kampo formulas were reported. We selected 2471 reports containing complete clinical data, including 210 reports on pseudoaldosteronism. In the pseudoaldosteronism group, 69.0% of patients were female, and 85.2% were ≥70 years old. The formulas most commonly associated with pseudoaldosteronism were Shakuyakukanzoto, Yokukansan, and Ryokeijutsukanto (ROR [95% confidence interval {CI}] = 18.3 [13.0–25.9], 8.1 [5.4–12.0], and 5.5 [1.4–21.9], respectively). Logistic analysis identified female sex (odds ratio [OR] [95% CI] = 1.7 [1.2–2.6]; *P* = 0.006), older age (≥70, 5.0 [3.2–7.8]; *P* < 0.001), low body weight (<50 kg, 2.2 [1.5–3.2]; *P* < 0.001), diuretics usage (2.1 [1.3–4.8]; *P* = 0.004), hypertension (1.6 [1.1–2.4]; *P* = 0.014), and dementia (7.0 [4.2–11.6]; *P* < 0.001) as pseudoaldosteronism-related factors. Additionally, the daily Glycyrrhiza dose (OR = 2.1 [1.9–2.3]; *P* < 0.001) and duration of administration (>14 days, OR = 2.8 [1.7–4.5]; *P* < 0.001) were associated with adverse events. We did not observe an interaction between aging and hypertension. Careful follow-up is warranted during long-term Glycyrrhiza-containing Kampo formula use in patients with multiple clinical factors for pseudoaldosteronism.

## Introduction

Kampo medicines are Japanese traditional treatments that have been developed for more than 1500 years [1]. To date, the Japanese Ministry of Health, Labour and Welfare has approved the use of 148 Kampo formulas under the national health insurance program, which promotes the integration of Kampo and Western medicines. Most doctors have experience prescribing Kampo formulas to patients [2, 3]. Moreover, many Japanese people are intensely interested in Kampo medicine [4]. However, Kampo formulas have been suggested to be associated with adverse events [5]. Therefore, understanding the risk factors associated with the adverse effects of Kampo formulas is crucial for improving their safe prescription and use.

Drug-induced pseudoaldosteronism is a well-known adverse event of Kampo medicine [5]. Pseudoaldosteronism is characterized by hypokalemia, metabolic alkalosis, and occasional hypertension. The pathophysiology of pseudoaldosteronism is hyporeninemic hypoaldosteronemia, whereas that of primary aldosteronism is hyporeninemic hyperaldosteronemia [6]. Past research indicated that glycyrrhizin, a principal component of Glycyrrhiza, is a potential cause of pseudoaldosteronism [7, 8]. Glycyrrhiza is a representative crude drug used in Western and Kampo medicines, foods, and drinks. Glycyrrhiza has various favorable effects in Kampo medicine, such as anti-inflammatory activity and beneficial effects on liver function [9, 10]. Therefore, more than 70% of Kampo formulas covered by Japanese health insurance contain Glycyrrhiza [11], exacerbating the difficulty in using glycyrrhizin-free Kampo medicines. Previous studies reported some concerns about the risk factors of pseudoaldosteronism, such as patients’ characteristics, concomitant drug usage, the daily Glycyrrhiza dosage, and the duration of Kampo formula usage [5, 12]. Moreover, recent studies reported a new metabolic system of Glycyrrhiza, suggesting some potential risks of Kampo-induced pseudoaldosteronism, such as hepatobiliary disorders and constipation [13]. However, limited research has utilized real-world databases to analyze the associated risk factors.

This study investigated the clinical factors linked to Kampo formula-induced pseudoaldosteronism using a data-mining approach.

## Materials and methods

### Database information

All data for this study were obtained from the Japanese Adverse Drug Event Report database (JADER) through the PMDA website [14]. JADER contains the following self-reported information: patient characteristics (gender, age in 10-year increments, body weight in 10-kg increments, and primary diseases), drugs (names, doses, and the causality of each drug such as “suspected drug,” “concomitant drug,” or “interacting drug”), and adverse events (types and outcomes).

JADER also registered information about reporting years, types of reports (trials, voluntary reports, others, and unknown), and types of reporters (doctors, pharmacists, medical personnel, consumers, and unknown). Multiple types of reporters can be involved with one patient in JADER. Therefore, we defined several types of reporters. 1. The category of doctors featured cases that included doctors as reporters. 2. The category of pharmacists featured cases that included pharmacists and excluded doctors as reporters. 3. The category of medical personnel featured cases that included medical personnel and excluded doctors and pharmacists as reporters. 4. The category of consumers featured cases that included consumers and excluded all medical personnel as reporters.

Notably, we could not obtain any data regarding exposure to specific drugs and information about patients who did not experience side effects after exposure to specific drugs from JADER. Therefore, it was impossible to calculate the absolute risk and incidence rate of pseudoaldosteronism attributable to specific Kampo formulas.

### Study design

First, we extracted all adverse events reported for 148 Kampo formulas categorized as suspected drugs from JADER. The selected Kampo formulas are covered by the Japanese national health insurance system (S1 Table). Second, we excluded cases with incomplete data. Third, we grouped patients according to the presence or absence of pseudoaldosteronism. We compared patients’ characteristics between the two groups and revealed the suspected Kampo formulas with reporting odds ratios (RORs) [15]. Finally, we conducted logistic analysis to assess the associated factors with pseudoaldosteronism caused by Kampo formulas.

### Definitions of adverse events and primary diseases

Our study defined all adverse events and primary diseases according to preferred terms (PTs) from the Medical Dictionary for Regulatory Activities/Japanese (MedDRA/J) version 25.1. The Standardized MedDRA Queries (SMQs) consist of PTs, including signs, symptoms, diagnoses, syndromes, physical findings, clinical laboratory data, and physiological analysis data. Pseudoaldosteronism was indicated by the following terms: pseudoaldosteronism (PT code: 10037113), blood potassium decreased (PT code: 10005724), and hypokalemia (PT code: 10021015) [16]. Additionally, we selected SMQs to define the primary diseases as follows: hypertension (SMQ code: 20000147), chronic kidney disease (SMQ code: 20000213), hepatobiliary disorder (SMQ code: 20000005, 20000118), dementia (SMQ code: 20000073), and constipation (PT code: 10010774).

### Definition of Glycyrrhiza dose

We obtained the Glycyrrhiza dose in each Kampo formula from the Standards of Reporting Kampo Products (STORK) website [17].

### ROR calculation

We calculated the ROR as follows: ROR = (A/B)/(C/D), where A represents reports including pseudoaldosteronism in patients who received the targeted Kampo formulas, B represents reports including pseudoaldosteronism in patients who did not receive the targeted Kampo formulas, C represents reports excluding pseudoaldosteronism in patients who received the targeted Kampo formulas, and D represents reports excluding pseudoaldosteronism in patients who did not receive the targeted Kampo formulas. We calculated the 95% confidence intervals (CIs) of RORs. We considered RORs with a lower 95% CI limit larger than 1 as meaningful signals for potential risks of pseudoaldosteronism [15].

### Statistical analyses

Categorical variables were analyzed using the chi-squared test or Fisher’s exact test. For continuous variables, data were analyzed using Student’s *t*-test. We evaluated the factors linked to pseudoaldosteronism via forced-entry logistic regression analysis of predefined co-variables. In our study, possible variables were predefined according to past reports as follows: age (≥70), gender, low body weight (<50 kg), primary diseases (chronic kidney disease, constipation, dementia, hepatobiliary disorders, hypertension, concomitant drug usage (diuretics, glycyrrhizinate, and corticosteroids), Glycyrrhiza dose, and duration of Kampo formula prescription [12–14, 16, 18]. Moreover, the interaction between variables was evaluated using multiplicative and additive scales [19, 20]. We assessed additive interactions using the relative excess risk due to interaction (RERI) and attributable proportion (AP) with their 95% CIs [21]. When the CIs of RERI and AP included 0, we concluded that the variables had no additive interactions. We calculate tolerance and variance inflation factor (VIF) to assess the multicollinearity between variables. VIF < 5 denoted independence of the variables [22]. The Hosmer–Lemeshow goodness-of-fit test was used to check the model’s fitness with *P* > 0.05 [23].

Continuous variables excluding the drug prescription period were presented as the mean and standard deviation. The drug prescription period was presented as the median and range. *P* < 0.05 indicated statistical significance. Statistical analyses were conducted using SPSS, version 29.0 (IBM, Armonk, NY, USA).

## Results

### Dataset preparation and patients’ characteristics

**Fig 1:**
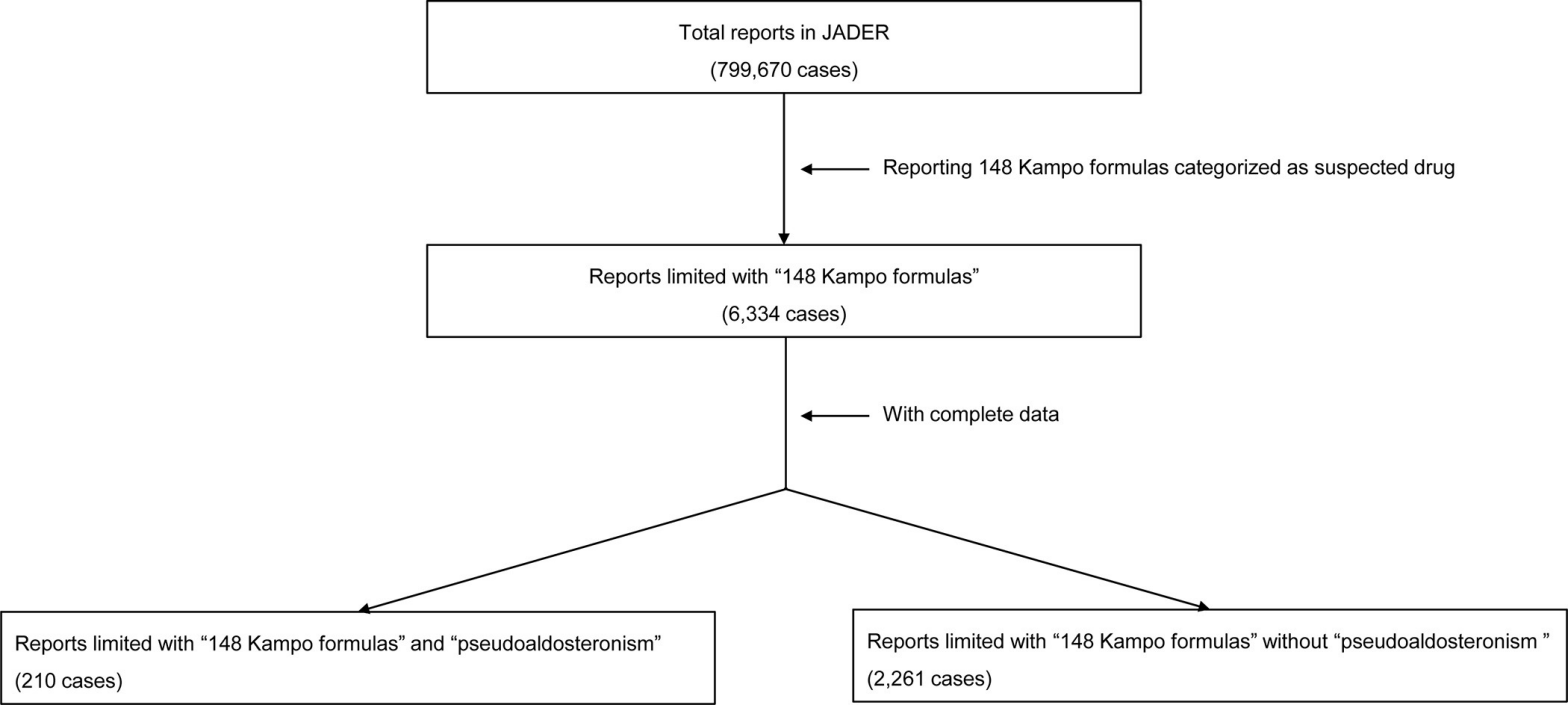
Flowchart in our study. JADER, the Japanese Adverse Drug Event Report database.

From April 2004 to November 2022, 799,670 adverse events were reported to JADER. Among them, we extracted 6334 cases reporting 148 Kampo formulas as the suspected drugs. After excluding reports with incomplete data, we included a final dataset of 2471 reports (210 pseudoaldosteronism cases and 2261 non-pseudoaldosteronism cases) in our study (Fig 1).

Table 1 presents patients’ characteristics in both groups. Female sex (69.0% vs. 59.6%, *P* = 0.007) and age ≥ 70 years (85.2% vs. 40.0%, *P* < 0.001) were significantly more common in the pseudoaldosteronism group than in the non-pseudoaldosteronism group. The most common age range in the pseudoaldosteronism group was 80–89. Additionally, low body weight (<50 kg) was more common in the pseudoaldosteronism group (54.3% vs. 31.9%, *P* < 0.001). Concomitant dementia (21.0% vs. 3.0%, *P* < 0.001) and hypertension (36.2% vs. 15.3%, *P* < 0.001) were more common in the pseudoaldosteronism group, whereas the incidence of chronic kidney disease, constipation, and hepatobiliary disorder did not differ between the groups. Concerning concomitant drugs, the frequency of diuretic usage was critically higher in the pseudoaldosteronism group (12.9% vs. 2.6%, *P* < 0.001). Contrarily, the usage of glycyrrhizinate-containing drugs did not significantly differ between the groups. We could not evaluate the oral usage of systemic corticosteroids because the administration method of corticosteroids was unclear in many cases. Notably, the Glycyrrhiza dose of Kampo formulas was significantly higher in the pseudoaldosteronism group (3.3 ± 2.0 g/day vs. 1.5 ± 1.3 g/day, *P* < 0.001). The median duration of Kampo formula prescription was 77.5 days (2–6287) in the pseudoaldosteronism group, versus 29.0 days (1–7767) in the non-pseudoaldosteronism group (*P* < 0.001).

**Table 1:** Patients’ characteristics.

	Pseudoaldosteronism group	Non-pseudoaldosteronism group	*P* value
	(*n* = 210)	(*n* = 2261)	
Gender, female, *n* (%)	145 (69.0)	1347 (59.6)	0.007
Age (years) *n* (%)			<0.001
<70	31 (14.8)	1356 (60.0)	
≥70	179 (85.2)	905 (40.0)	
Body weight (kg) *n* (%)			<0.001
<50	114 (54.3)	722 (31.9)	
≥50	96 (45.7)	1539 (68.1)	
Primary disease, *n* (%)			
Chronic kidney disease	6 (2.9)	38 (1.7)	0.165
Constipation	11 (5.2)	92 (4.1)	0.417
Dementia	44 (21.0)	67 (3.0)	<0.001
Hepatobiliary disorder	10 (4.8)	97 (4.3)	0.748
Hypertension	76 (36.2)	345 (15.3)	<0.001
Concomitant oral drug, *n* (%)			
Diuretics	27 (12.9)	58 (2.6)	<0.001
Glycyrrhizinate-containing drugs	1 (0.5)	7 (0.3)	0.509
Dose of Glycyrrhiza, mean [SD] (g/day)	3.3 [2.0]	1.5 [1.3]	<0.001
Prescription period of Kampo formulas, median [range] (days)	77.5 [2–6287]	29.0 [1–7767]	<0.001

SD, standard deviation.

### Evaluation of years and types of extracted cases

**Fig 2:**
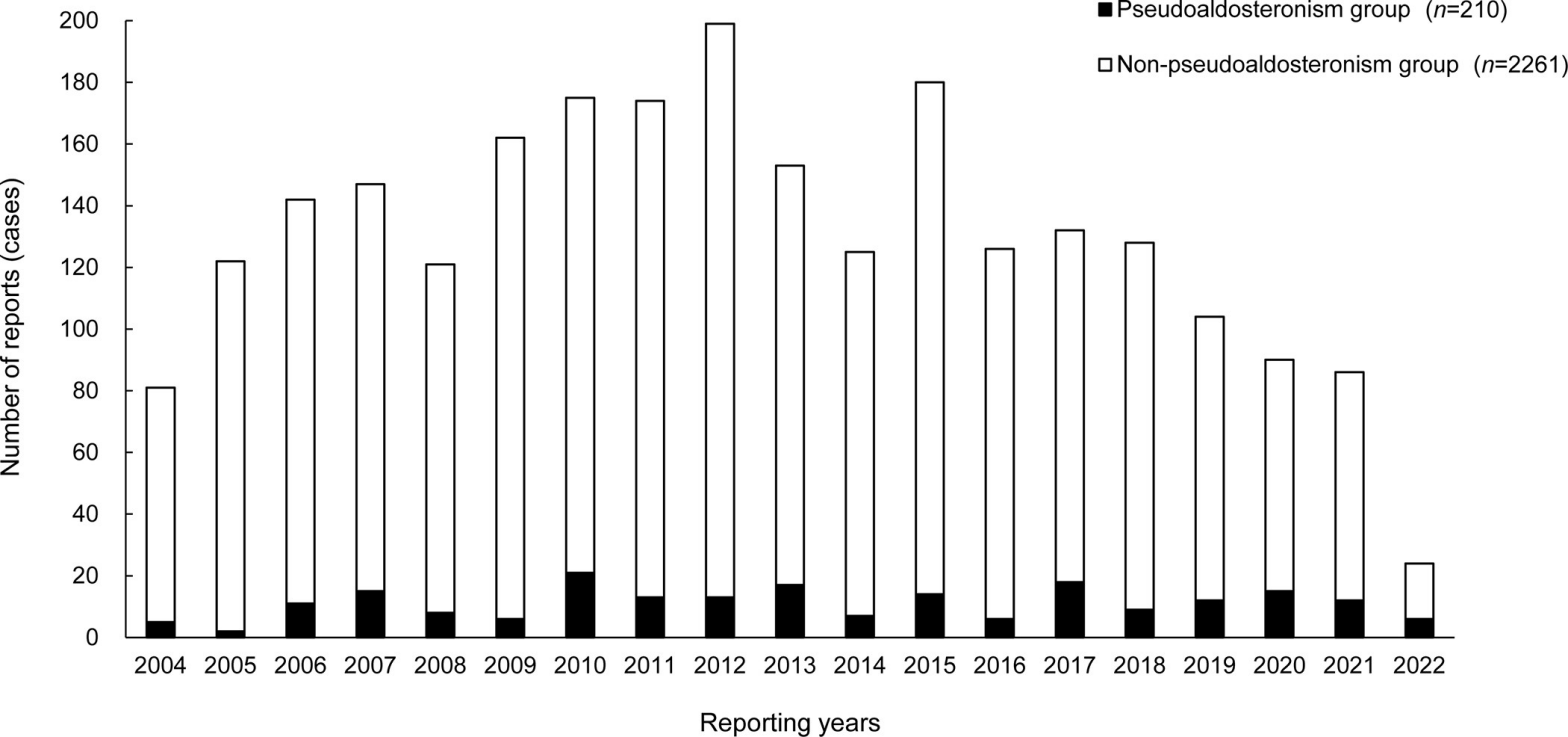
Annual changes in the number of reports of Kampo formula-induced adverse events. Black bar, the pseudoaldosteronism group; white bar, the non-pseudoaldosteronism group.

We summarized data about the years, types of reports, and reporters (Fig 2, Table 2). Extracted cases of adverse events were steadily reported each year in both groups. Moreover, most cases were voluntarily reported cases in both groups. Conversely, the types of reporters differed between the pseudoaldosteronism and non-pseudoaldosteronism groups. In particular, the proportion of reports from doctors was smaller in the pseudoaldosteronism group.

**Table 2:** Type of reports and reporters.

	Pseudoaldosteronism group (*n* = 210)	Non-pseudoaldosteronism group (*n* = 2261)	*P* value
Types of reports, *n* (%)			0.176
Trials	8 (3.8)	77 (3.4)	
Voluntary reports	181 (86.2)	1995 (88.2)	
Others	20 (9.5)	188 (8.3)	
Unknown	1 (0.5)	1 (0.0)	
Types of reporters, *n* (%)			<0.001
Doctors	153 (72.9)	2077 (84.1)	
Pharmacists	53 (25.2)	260 (10.5)	
Medical personnel	3 (1.4)	85 (3.4)	
Consumers	0 (0.0)	1 (0.0)	
Unknown	1 (0.5)	48 (1.9)	

The category of doctors comprised cases that included doctors as reporters. The category of pharmacists comprised cases that included pharmacists and excluded doctors as reporters. The category of medical personnel comprised cases that included medical personnel and excluded doctors and pharmacists as reporters. The category of consumers comprised cases that included consumers and excluded all medical personnel as reporters.

### Kampo formulas most frequently suspected to cause pseudoaldosteronism

**Fig 3:**
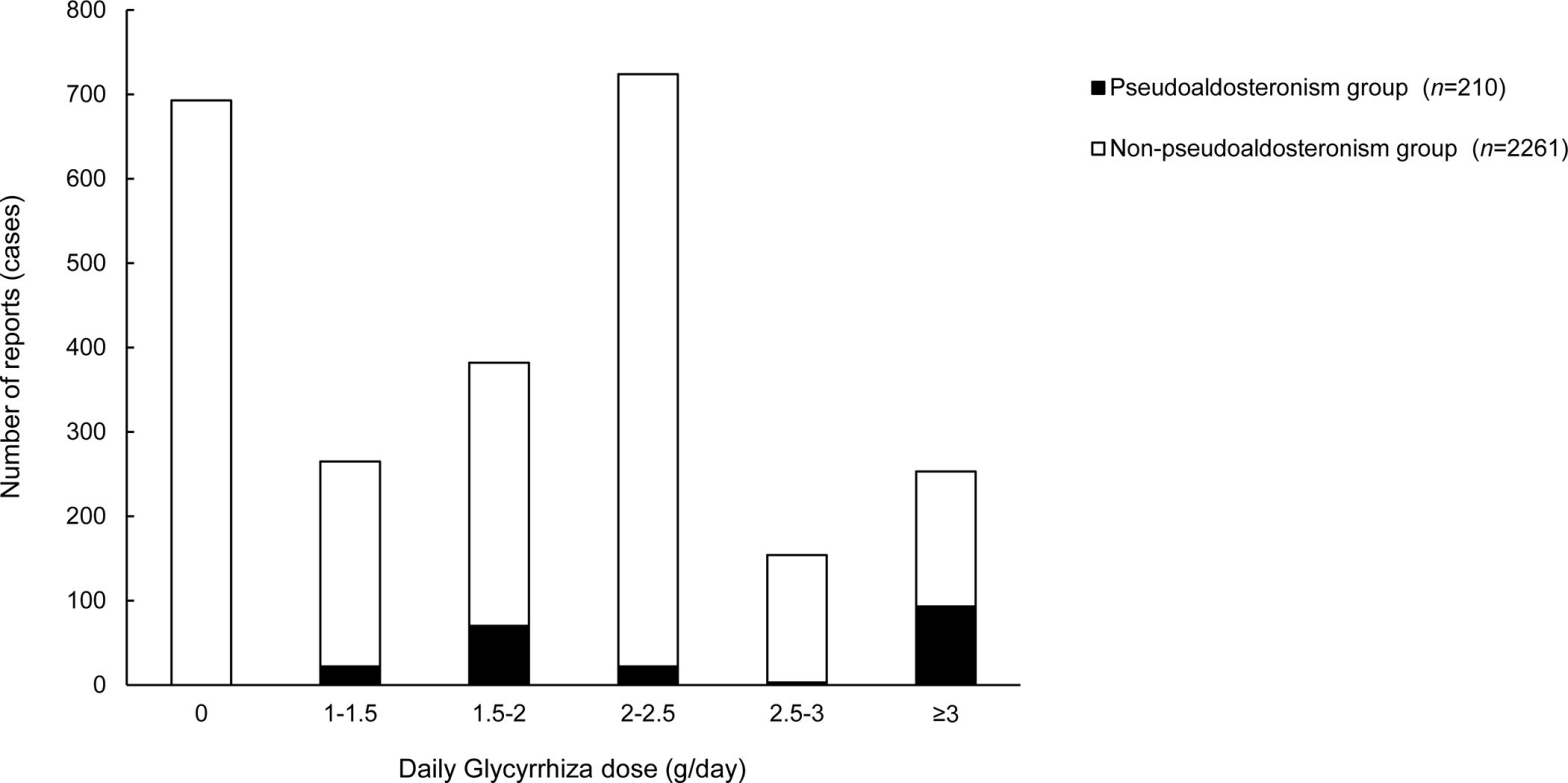
Distribution of daily Glycyrrhiza dosage in Kampo formulas. Black bar, the pseudoaldosteronism group; white bar, the non-pseudoaldosteronism group.

All extracted Kampo formulas regarded as suspected drugs for pseudoaldosteronism are presented in Table 3. Among 210 cases of pseudoaldosteronism, Shakuyakukanzoto was the most frequently suspected drug (42.9%), followed by Yokukansan (22.4%) and Rikkunshito (5.7%). Additionally, the highest RORs recorded were for Shakuyakukanzoto (18.3 [95% CI = 13.0–25.9]), Yokukansan (8.1 [95% CI = 5.4–12.0]), and Ryokeijutsukanto (5.5 [95% CI = 1.4–21.9]). In our study, all suspected Kampo formulas contained Glycyrrhiza. Fig 3 presents the distribution of the number of patients by the daily dose of Glycyrrhiza. All pseudoaldosteronism cases involved Glycyrrhiza-containing Kampo formulas, whereas 30.7% of cases of non-pseudoaldosteronism did not involve Glycyrrhiza. In 44.2% of patients in the pseudoaldosteronism group, the daily Glycyrrhiza dosage was 3 g or more.

**Table 3:** List of suspected Kampo formulas.

Kampo formulas	Glycyrrhiza dose (g/day)	Total (*n* = 2471)	Pseudoaldosteronism group (*n* = 210)	Non-pseudoaldosteronism group (*n* = 2261)	RORs in Kampo medicine
Shakuyakukanzoto	5.0–6.0	179	90	89	18.3 [13.0–25.9]
Yokukansan	1.5	125	47	78	8.1 [5.4–12.0]
Rikkunshito	1.0–1.5	80	12	68	2.0 [1.0–3.7]
Hochuekkito	1.5	58	10	48	2.3 [1.2–4.6]
Juzentaihoto	1.0–1.5	25	6	19	3.5 [1.4–8.8]
Kakkonto	2.0	73	5	68	0.8 [0.3–2.0]
Saireito	2.0	183	5	178	0.3 [0.1–0.7]
Boiogito	1.5	29	5	24	2.3 [0.9–6.0]
Hangeshashinto	2.5–3.0	101	3	98	0.3 [0.1–1.0]
Bakumondoto	2.0	61	3	58	0.6 [0.2–1.8]
Ryokeijutsukanto	2.0	9	3	6	5.5 [1.4–21.9]
Ninjin’yoeito	1.0	11	2	9	2.4 [0.5–11.2]
Daiokanzoto	1.0–2.0	9	2	7	3.1 [0.6–15.0]
Saibokuto	2.0	58	1	57	0.2 [0.0–1.3]
Keishikashakuyakuto	2.0	7	1	6	1.8 [0.2–15.0]
Junchoto	1.5	26	1	25	0.4 [0.1–3.1]
Kanzoto	8.0	1	1	0	Incalculable
Chotosan	1.0	6	1	5	2.2 [0.3–18.6]
Seishinrenshiin	1.5–2.0	49	1	48	0.2 [0.0–1.6]
Keishikajutsubuto	2.0	3	1	2	5.4 [0.5–60.0]
Kikyoto	3.0	4	1	3	3.6 [0.4–34.8]
Maoto	1.5	36	1	35	0.3 [0.0–2.2]
Tokishigyakukagoshuyushokyoto	2.0	12	1	11	1.0 [0.1–7.6]
Yokukansankachimpihange	1.5	11	1	10	1.1 [0.1–8.5]
Ninjinto	3.0	5	1	4	2.7 [0.3–24.3]
Shigyakusan	1.5	1	1	0	Incalculable
Keishito	2.0	4	1	3	3.6 [0.4–34.8]
Anchusan	1.0–2.0	3	1	2	5.4 [0.5–59.9]
Makyokansekito	2.0	7	1	6	1.8 [0.2–15.0]
Kososan	1.0–1.5	6	1	5	2.2 [0.3–18.6]

RORs were described with their 95% confidence intervals. RORs, relative odds ratio.

### Clinical factors associated with Kampo formula-induced pseudoaldosteronism

Finally, we performed logistic regression analysis with the forced-entry approach to identify the factors related to Kampo formula-induced pseudoaldosteronism (Table 4). Our analysis revealed that female sex (odds ratio [OR] = 1.7 [95% CI = 1.2–2.6], *P* = 0.006), age ≥ 70 (OR = 5.0 [95% CI = 3.2–7.8], *P* < 0.001), low body weight (OR = 2.2 [95% CI = 1.5–3.2], *P* < 0.001), and diuretics use (OR = 2.1 [95% CI = 1.3–4.8], *P* = 0.004) were independently linked to Kampo formula-induced pseudoaldosteronism. In addition, significant associations were observed in patients with dementia (OR = 7.0 [95% CI = 4.2–11.6], *P* < 0.001) and hypertension (OR = 1.6 [95% CI = 1.1–2.4], *P* = 0.014), whereas chronic kidney disease, constipation, and hepatobiliary disorder were not associated with pseudoaldosteronism. Moreover, a larger daily Glycyrrhiza dose (OR = 2.1 [95% CI = 1.9–2.3], *P* < 0.001) and long prescription periods (>14 days; OR = 2.8 [95% CI = 1.7–4.5], *P* < 0.001) were significantly linked to Kampo formula-induced pseudoaldosteronism. The VIF exceeded 1 in all regression analyses, which supported the robustness of our findings. The result of the Hosmer–Lemeshow goodness-of-fit test was not significant (*P* = 0.250). Moreover, we checked for multiplicative and additive interaction between age and hypertension (Table 5). In our analysis, the multiplicative and additive interaction indices did not indicate critical significance between the two co-variables (OR [95% CI]: multiplicative score, 0.5 [0.2–1.2]; RERI, 5.2 [−1.0–13.6]; and AP, 0.3 [−0.1–0.5]).

**Table 4:** Logistic regression model for Kampo formula-induced pseudoaldosteronism.

Co-variables	Odds ratio	95% confidence interval		*P* value	Multicollinearity statistics	
		Lower	Upper		Tolerance	VIF
Female	1.7	1.2	2.6	0.006	0.9	1.1
Aging (>70 years)	5.0	3.2	7.8	<0.001	0.9	1.2
Low body weight (<50 kg)	2.2	1.5	3.2	<0.001	0.9	1.2
Diuretics	2.1	1.3	4.8	0.004	0.9	1.1
Hypertension	1.6	1.1	2.4	0.014	0.9	1.1
Dementia	7.0	4.2	11.6	<0.001	0.9	1.1
Glycyrrhiza dose (g/day)	2.1	1.9	2.3	<0.001	1.0	1.0
Prescription period of Kampo formulas (>14 days)	2.8	1.7	4.5	<0.001	1.0	1.0

VIF, variance inflation factor

**Table 5:** Multiplicative and additive interactions between age and dementia.

Older	Hypertension	Odds ratio	95% confidence interval	
(>70 years)			Lower	Upper
No	No	1 [Reference]		
No	Yes	3.9	1.8	8.7
Yes	No	9.1	5.7	14.5
Yes	Yes	17.2	10.4	28.5
Multiplicative scale		0.5	0.2	1.2
RERI		5.2	-1.0	13.6
AP		0.3	-0.1	0.5

RERI, relative excess risk due to interaction; AP, attributable proportion

## Discussion

This was the first study to investigate the real-world factors associated `with Kampo formula-induced pseudoaldosteronism using a data-mining approach, and several clinical factors, including hypertension, the daily Glycyrrhiza dosage, and the duration of Kampo formula administration, were cited as attentional factors.

Previous research indicated that glycyrrhizin, the chief component of Glycyrrhiza, and its metabolites cause pseudoaldosteronism by suppressing 11β-hydroxysteroid dehydrogenase type 2 (11β-HSD2) in renal distal tubular epithelial cells [13, 24]. We identified concomitant hypertension as an independent factor linked to Kampo formula-induced pseudoaldosteronism. 11β-HSD2 inactivates glucocorticoid and enhances the ability of aldosterone to bind mineralocorticoid receptors in renal distal tubule cells. Deficiency of HSD11B2, the gene encoding 11β-HSD2, causes apparent mineralocorticoid excess syndrome, a Mendelian hypertensive disorder [25]. Additionally, a previous basic study reported that renal 11β-HSD2 inactivity is associated with the onset of hypertension [26]. This information supports our result that hypertension is an important factor for Kampo formula-induced pseudoaldosteronism. Our results also identified concomitant dementia as an independent factor associated with Kampo formula-induced pseudoaldosteronism. Few studies have assessed the relationship between dementia and pseudoaldosteronism, although some case reports warned of the delayed recognition of side effects attributable to dementia [7, 16]. Further investigation is warranted to elucidate the mechanism of the relationship between pseudoaldosteronism and dementia.

Conversely, concomitant chronic kidney disease was not identified as a significant factor in our study. Although a prior study reported that 11β-HSD2 activity was decreased in pediatric patients with chronic kidney disease, another study found that the impact of kidney injury on pseudoaldosteronism decreased with age [27]. Because most patients in the pseudoaldosteronism group were elderly, the age distribution in our study could have affected the result. The rates of hepatobiliary disorders and constipation did not significantly differ between the pseudoaldosteronism and non-pseudoaldosteronism groups. These variables have been reported as risk factors for Kampo medicine-induced pseudoaldosteronism because glycyrrhizin is metabolized by the gut biota and hepatic enzymes [28, 29]. Future investigations should clarify this discrepancy.

As found in past reports [16, 30, 31], our logistic regression analysis confirmed that older age, low body weight, female sex, and diuretics usage are attentional factors for Kampo formula-induced pseudoaldosteronism. Renal 11β-HSD2 activity is decreased among older patients [32]. In addition, hypoalbuminemia can inhibit renal 11β-HSD2 activity because of the low binding rate between glycyrrhizin metabolites and serum albumin [11, 30]. These reports proposed that older patients with malnutrition should be warned of the risks of pseudoaldosteronism. There was little evidence indicating the relationship between gender and 11β-HSD2. Future research is needed to clarify this issue.

Our results also confirmed that the daily Glycyrrhiza dosage was independently related to pseudoaldosteronism. Past studies announced that daily Glycyrrhiza doses of 2.5 g or higher increased the risk of Kampo-induced pseudoaldosteronism [12, 16, 31]. In contrast to these studies, Yokukansan and Ryokeijutsukanto, which had second and third highest RORs in our study, both contain less than 2.5 g of Glycyrrhiza.

Notably, we identified that more than 14 days of treatment with Kampo formulas was significantly associated with pseudoaldosteronism. Previous research mentioned that the risk of pseudoaldosteronism increases with an increasing duration of treatment. However, similar to our findings, past studies reported various durations of Kampo formula treatment that increased the risk of pseudoaldosteronism [33]. We set the cutoff as 14 days because 2 weeks of glycyrrhetic acid treatment were required to stably suppress renal 11β-HSD2 activity [13, 34]. Meanwhile, 40% of reports indicated that pseudoaldosteronism developed within 3 months of treatment [14]. Fourteen days of treatment with Kampo formulas could be a benchmark for inducing pseudoaldosteronism.

This study had several limitations. First, the data only consisted of self-reported adverse events, which can be subject to underreporting, incomplete, and limited information on the clinical background of the patients. Therefore, we could not calculate the incidence rate of pseudoaldosteronism for each Kampo formula. Some patients’ characteristics, such as dementia and constipation, had potential risks of inaccurate diagnosis or underreporting. On the contrary, selection bias might have reduced the number of mild pseudoaldosteronism reports and resulted in the underestimation of adverse effects. Second, we could not calculate RORs in comparison with patients who developed pseudoaldosteronism in the absence of Kampo formula treatment because of the low number of reports. Notably, the RORs in our study had some uncertainty, and they might differ from the absolute risk for each Kampo formula because of the properties of the database. Third, we could not evaluate the risk of concomitant corticosteroid usage, although it was considered a risk factor for Kampo formula-induced pseudoaldosteronism [31]. Fourth, the study cohort was limited to the Japanese population, limiting the generalizability of the findings to other populations. Future studies should consider prospective designs and larger sample sizes to validate the identified risk factors and explore additional factors associated with Kampo formula-induced pseudoaldosteronism.

## Conclusions

We revealed real-world associated factors with Kampo formula-induced pseudoaldosteronism. Among the identified related factors, concomitant hypertension was meaningful. When Glycyrrhiza-containing Kampo formulas are prescribed for more than 14 days to patients with multiple attentional factors, careful follow-up is required to prevent the onset of pseudoaldosteronism.

## Supporting information

S1 Table Kampo formulas covered by Japanese health insurance. (XLSX)
